# Novel Arenavirus Sequences in *Hylomyscus* sp. and *Mus (Nannomys) setulosus* from Côte d'Ivoire: Implications for Evolution of Arenaviruses in Africa

**DOI:** 10.1371/journal.pone.0020893

**Published:** 2011-06-09

**Authors:** David Coulibaly-N'Golo, Bernard Allali, Stéphane K. Kouassi, Elisabeth Fichet-Calvet, Beate Becker-Ziaja, Toni Rieger, Stephan Ölschläger, Hernri Dosso, Christiane Denys, Jan ter Meulen, Chantal Akoua-Koffi, Stephan Günther

**Affiliations:** 1 Laboratoire des Arbovirus/Entérovirus, Institut Pasteur de Côte d'Ivoire, Abidjan, Côte d'Ivoire; 2 Department of Virology, Bernhard-Nocht-Institute for Tropical Medicine, Hamburg, Germany; 3 Département Systématique et Evolution, Museum National d'Histoire Naturelle, Paris, France; 4 Centre de Recherche en Ecologie, Universite d'Abobo-Adjame, Abidjan, Côte d'Ivoire; 5 Institute of Virology, Philipps University, Marburg, Germany; 6 Merck Research Laboratories, West Point, Pennsylvania, United States of America; University of Pretoria, South Africa

## Abstract

This study aimed to identify new arenaviruses and gather insights in the evolution of arenaviruses in Africa. During 2003 through 2005, 1,228 small mammals representing 14 different genera were trapped in 9 villages in south, east, and middle west of Côte d'Ivoire. Specimens were screened by pan-Old World arenavirus RT-PCRs targeting S and L RNA segments as well as immunofluorescence assay. Sequences of two novel tentative species of the family *Arenaviridae*, Menekre and Gbagroube virus, were detected in *Hylomyscus* sp. and *Mus (Nannomys) setulosus*, respectively. Arenavirus infection of *Mus (Nannomys) setulosus* was also demonstrated by serological testing. Lassa virus was not found, although 60% of the captured animals were *Mastomys natalensis*. Complete S RNA and partial L RNA sequences of the novel viruses were recovered from the rodent specimens and subjected to phylogenetic analysis. Gbagroube virus is a closely related sister taxon of Lassa virus, while Menekre virus clusters with the Ippy/Mobala/Mopeia virus complex. Reconstruction of possible virus–host co-phylogeny scenarios suggests that, within the African continent, signatures of co-evolution might have been obliterated by multiple host-switching events.

## Introduction

*Arenaviridae* are negative strand RNA viruses hosted by rodents of the superfamily Muroidea. Previous comparisons of arenavirus phylogeny with rodent host phylogeny provided several examples in which virus–host co-speciation was potentially occurring during evolution [1]. Therefore, current hypotheses assume that arenaviruses share a long-term evolutionary relationship with their rodent hosts, an evolutionary model called co-evolution. Africa is home to various Old World arenaviruses (OWA) [2], [3], [4], [5], [6], [7], [8], of which Lassa and Lujo virus cause hemorrhagic fever in humans [9], [10], [11]. Lassa virus is endemic in the countries of Nigeria, Liberia, Sierra Leone, and Guinea [1], [9], [12] and was recently also detected in Mali [13], [14]. Whether OWAs including Lassa virus are endemic in Côte d'Ivoire is not known. Seroepidemiological studies and an imported case of Lassa fever indicate that arenaviruses circulate somewhere in the region comprising Ghana, Côte d'Ivoire, and Burkina Faso [15], [16]. We conducted a systematic search for OWAs in wildlife in Côte d'Ivoire. While Lassa virus was not found, two novel arenaviruses were identified and characterized molecularly. The sequence data were used to gather new insights in the evolution of arenaviruses in Africa.

## Results

**Figure 1:**
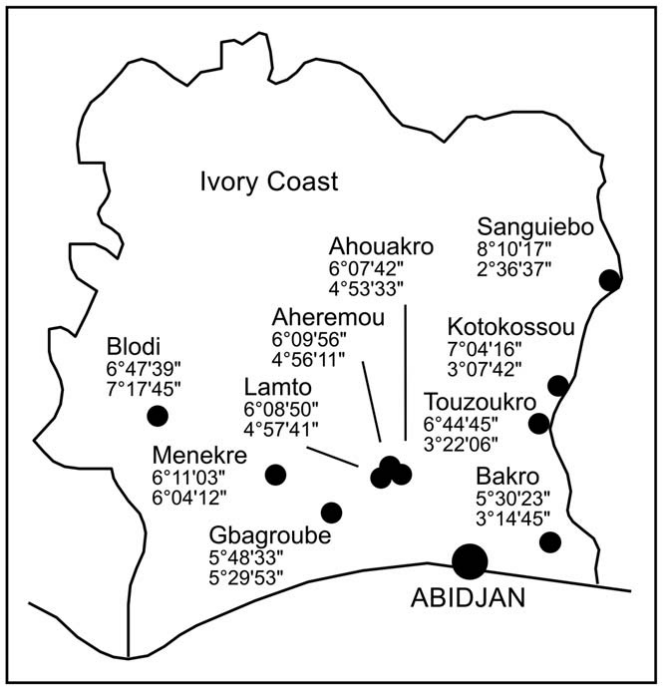
Map of Côte d'Ivoire showing the capture sites. Latitude North and longitude West coordinates are given below the name of the villages.

During 2003 through 2005, 1,228 small mammals representing 14 different genera were trapped in 9 villages in south, east, and middle west of Côte d'Ivoire (Table 1 and Fig. 1). Arenavirus screening was performed by using two pan-OWA RT-PCRs targeting glycoprotein precursor (GPC) and large (L) gene, respectively [17]. Six out of 1,228 samples tested positive in both assays (Table 1). None of them was positive for Lassa virus. Three of the positive samples (CIV839, CIV843, and CIV1227) originated from *Hylomyscus* sp. captured in Menekre, Prefecture Gagnoa, and three samples (CIV608, CIV674, and CIV1290) originated from *Mus (Nannomys)* sp. captured in Gbagroube, Prefecture Divo. The six virus RNA-positive animals were genotyped by sequencing the mitochondrial cytochrome B and 16S rRNA genes. Phylogenetic analysis indicates that the rodents from Menekre presumably constitute a new *Hylomyscus* species (details to be reported elsewhere), while the rodents from Gbagroube are *Mus (Nannomys) setulosus*. The virus RNA-positive *Hylomyscus* species had been trapped in cocoa farm and forest, and the positive *Mus (Nannomys) setulosus* in banana plantations and forest. Both are usually forest species living in zones with high rainfall. Additional testing of all *Hylomyscus* sp. (n = 3) and all *Mus (Nannomys) setulosus* (n = 47) that were negative in the initial screening using RT-PCRs specific for the new virus sequences did not increase the number of positive animals. Thus, virus prevalence was 100% (3/3) in *Hylomyscus* sp. from Menekre and 12% (3/25) in *Mus (Nannomys) setulosus* from Gbagroube (Table 1).

**Table 1:** Small mammals from Côte d'Ivoire tested by pan-Old World arenavirus RT-PCR, Lassa virus-specific RT-PCR, and Old World arenavirus immunofluorescence assay^a^.

	No. tested (RT-PCR-positive;antibody-positive) per sampling site^c^									
Genus or species^b^	Ah	Ba	Bl	Gb	Ko	La	Me	Sa	To	Total
*Crocidura* spp.	11	4	5	15 (0;1)	4	2	12	6	2	61 (0;1)
*Dasymys* sp.	1	–	–	–	–	–	–	–	–	1
*Gerbilliscus* sp.	5	–	–	–	1	10	–	1	–	17
*Grammomys* sp.	1	–	–	–	–	–	–	–	–	1
*Graphiurus* sp.	–	–	1	1	–	–	–	–	–	2
***Hylomyscus*** ** sp.**	–	–	1	–	–	2	**3 (3;0)** d	–	–	6 (3;0)
*Lemniscomys* spp.	15	2	–	–	1	8	–	1	–	27
*Lophuromys sikapusi*	17 (0;2)	4	8	9	4	–	7	–	9	58 (0;2)
*Malacomys* sp.	–	–	1	–	–	3	1	–	–	5
*Mastomys erythroleucus*	2	–	–	1	–	1	–	3	–	7
*Mastomys natalensis* e	221 (0;1)	–	97	164	12	–	104 (0;2)	128 (0;2)	3	729 (0;5)
*Mus musculus*	–	–	–	–	–	–	4	–	–	4
*Mus (Nannomys) baoulei* f	–	–	–	–	–	2	–	1	–	3
*Mus (Nannomys) mattheyi* f	–	–	–	–	–	–	–	1	–	1
*Mus (Nannomys) minutoides* f	3	4	12 (0;2)	15	1 (0;1)	2	3 (0;1)	2	7	49 (0;4)
*Mus (Nannomys) musculoides* f	–	–	–	–	–	–	–	1	–	1
***Mus (Nannomys) setulosus*** f	–	5	2	**25 (3;7)** g	2	3	–	–	13	50 (3;7)
*Praomys* (formerly *Myomys*) *daltoni*	–	1	–	2	42	–	–	2	27	74
*Praomys* spp.	5	1	4	4	9	4	–	3	2	32
*Rattus rattus*	14	–	6	12	–	–	16	–	–	48
*Uranomys ruddi*	46	–	–	–	1	4	–	1	–	52
Total	341 (0;3)	21	137 (0;2)	248 (3;8)	77 (0;1)	41	150 (3;3)	150 (0;2)	63	1228 (6;19)

a Collections with RT-PCR-positive samples as well as the respective animals are indicated in boldface.

b Taxonomic classification is based on morphological field data, if not otherwise indicated.

c The number of positive animals is given in parentheses in the order: RT-PCR-positive; immunofluorescence assay-positive. If none of the animals was positive in either test, only the number of tested animals is indicated. Sampling sites: Ah, Ahouakro; Ba, Bakro; Bl, Blodi; Gb, Gbagroube; Ko, Kotokossou; La, Lamto; Me, Menekre; Sa, Sanguiebo; To, Touzoukro.

d Samples CIV839, CIV843, and CIV1227 tested positive in pan-Old World arenavirus (OWA) large (L) and glycoprotein precursor (GPC) gene RT-PCRs.

e The mitochondrial cytochrome B gene of a representative subset of 73 animals, including the antibody-positive animals, from Ahouakro, Blodi, Gbagroube, Kotokossou, Menekre, and Sanguiebo was sequenced. All animals sequenced were genetically confirmed to be *Mastomys natalensis*.

f Animals were classified by sequencing the cytochrome B gene. Cytogenetic, molecular, and morphometrical analysis has been published for a subset [47].

g Samples CIV608, CIV674, and CIV1290 tested positive in pan-OWA L and GPC gene RT-PCRs. Seven further animals were positive for anti-OWA IgG antibodies.

Sequencing the GPC and L gene PCR fragments (∼960 and 340 nucleotides, respectively) revealed that all strains from *Hylomyscus* sp. constitute a new arenavirus, called Menekre virus, while all strains from *Mus (Nannomys) setulosus* constitute another new arenavirus, called Gbagroube virus. Attempts to isolate the viruses in cell culture or mice were not successful (see Materials and Methods for details). Therefore, the complete 3.5-kb S RNA segment of the viruses was sequenced directly from the rodent specimens using pan-OWA PCR primers targeting the nucleoprotein (NP) gene and a set of virus-specific primers. Full-length S RNA sequences (excluding the conserved 19 nucleotides at the termini) were obtained for Menekre virus strain CIV1227 (3423 nucleotides) and Gbagroube virus strain CIV608 (3347 nucleotides), and partial NP sequences were obtained for Menekre virus strains CIV839 and CIV843 and Gbagroube virus strains CIV674 and CIV1290 (∼1200 nucleotides). Virus and associated host sequences reported in this article have been submitted to GenBank (Table 2).

**Table 2:** Sequences generated in this study.

		Virus genesGenBank accession no.				Host genesGenBank accession no.	
Specimen	Virus	GPC	NP	L	Host	Cyt-B	16S rRNA
CIV608	Gbagroube	GU830848^a^	GU830848^a^	GU830849	*Mus setulosus*	GU830865	GU830864
CIV674	Gbagroube	GU830850	GU830851	GU830852	*Mus setulosus*	GU830867	GU830866
CIV1290	Gbagroube	GU830853	GU830854	GU830855	*Mus setulosus*	GU830869	GU830868
CIV839	Menekre	GU830856	GU830857	GU830858	*Hylomyscus* sp.	GU830845	GU830844
CIV843	Menekre	GU830859	GU830860	GU830861	*Hylomyscus* sp.	GU830871	GU830870
CIV1227	Menekre	GU830862^a^	GU830862^a^	GU830863	*Hylomyscus* sp.	GU830847	GU830846

a Complete 3.5-kb S RNA sequences. All other virus sequences are partial.

To provide additional evidence for the circulation of arenaviruses in Côte d'Ivoire, all PCR-negative animals captured in the country were tested for the presence of anti-OWA antibodies by immunofluorescence assay (Table 1). Lassa virus was used as an antigen due to its known cross-reactivity with immune sera from animals infected with other OWAs [3], [6], [18]. While the remaining three *Hylomyscus* sp. were antibody-negative, 7 of 50 *Mus (Nannomys) setulosus* were positive for anti-OWA IgG antibodies. All seven animals had been captured in Gbagroube in March, August, and November 2005, resulting in an overall seroprevalence of 28% (7 of 25) in this area. Thus, serological testing confirms arenavirus infections in *Mus (Nannomys) setulosus* in Gbagroube. In addition, 12 animals of other species, including *Crocidura* spp. (1/61), *Lophuromys sikapusi* (2/58), *Mastomys natalensis* (5/729), and *Mus (Nannomys) minutoides* (4/49), were seropositive (Table 1).

Genetic distance analysis revealed that Gbagroube virus is closely related to Lassa virus (Table 3). The minimum amino acid difference (uncorrected *p*-distance) to Lassa virus in NP (14.1%) slightly exceeds the maximum intra-species genetic distance in OWA complex (12.1%). However, the minimum distance in GPC (9.8%) is smaller than the maximum intra-species difference within the Mopeia and lymphocytic choriomeningitis virus (LCMV) clades (11.9% and 20.9%, respectively). Menekre virus is virtually equidistant to Lassa, Gbagroube, Mopeia, Mobala, and Ippy virus (25.4–29.7% in NP and 22.2–25.5% in GPC) (Table 3). We consider Gbagroube and Menekre virus new species of the family *Arenaviridae*, since both exceed the proposed species-differentiating cut-off value of 12% amino acid difference in NP [19] and are associated with specific rodents that have so far not been described as arenavirus host species. The fact that non-identical sequences of Menekre virus and Gbagroube virus were found in three *Hylomyscus* sp. and three *Mus (Nannomys) setulosus*, respectively, and in none of the other animal species strongly suggests that the virus sequences were recovered from the natural host species. The high prevalence of anti-OWA antibodies in *Mus (Nannomys) setulosus* from the Gbagroube village lend further support to this conclusion.

**Table 3:** Genetic distance of Gbagroube and Menekre virus to other Old World arenaviruses.

	% mean (range) uncorrected *p* amino acid distance			
	Gbagroube virus		Menekre virus	
Virus	NP	GPC	NP	GPC
Lassa^a^	15.2 (14.1–16.1)	10.6 (9.8–11.6)	25.6 (24.7–26.8)	24.6 (23.7–25.5)
Gbagroube	–	–	25.4	25.5
Mopeia^a^	27.4 (27.4–27.5)	22.7 (22.2–23.1)	26.6 (26.4–27.0)	22.2 (21.5–23.3)
Mobala	28.8	21.8	28.9	25.3
Menekre	25.4	25.5	–	–
Ippy	29.5	29.6	29.7	24.4
Merino Walk	30.9	31.6	31.5	30.1
LCM^a^^,^ b	37.2 (36.2–38.3)	42.8 (41.4–45.7)	38.1 (37.3–39.0)	43.4 (42.1–44.7)
Lujo	44.0	61.4	44.9	62.9

a The maximum intra-species genetic distance (% uncorrected *p* amino acid distance) for Lassa, Mopeia, and lymphocytic choriomeningitis (LCM) virus clade is 11.6, 12.1, and 11.3 in NP; and 9.2, 11.9, and 20.9 in GPC, respectively.

b Including strains hosted by *Apodemus sylvaticus*.

**Figure 2:**
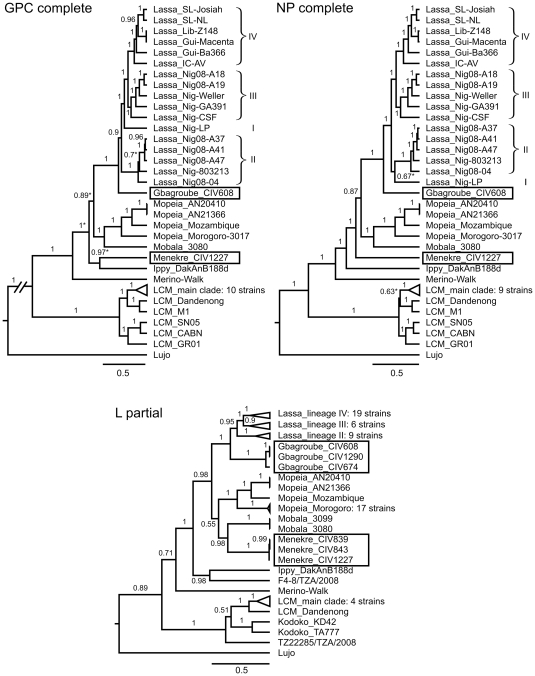
Phylogenetic analysis of Old World arenaviruses including Gbagroube and Menekre virus. Phylogenies were inferred by BEAST software based on nucleotide sequences of complete GPC and NP gene, and partial L gene. Posterior values are indicated on the branches. For GPC and NP genes, the phylogeny was verified by using PhyML software (trees not shown). Branches which are not or poorly supported by PhyML (bootstrap values <0.5) are indicated with an asterisk; all other branches are supported with bootstrap values >0.5. For clarity of presentation, some distal branches were collapsed. The LCM virus main clade includes strains CH-5692, CH-5871, Armstrong, WE, Traub, MX, Pasteur, UBC-aggressive, and Marseille 12. The origin of Lassa virus strains is indicated by a prefix: SL, Sierra Leone; LIB, Liberia; GUI, Guinea; IC, Ivory Coast; NIG, Nigeria. Lassa virus lineages are indicated by roman numerals. The complete list of taxa with GenBank accession numbers is provided in Table S1.

In the phylogeny, Gbagroube virus has a sister relationship with Lassa virus in GPC, NP, and L gene trees (Fig. 2), which is consistent with the small genetic distance between both viruses. The most recent common ancestor of the two clades is supported by posterior and bootstrap values. The position of Menekre virus in the OWA trees is ambiguous, consistent with the genetic equidistance to all viruses except Merino Walk, LCM, and Lujo virus. It shows a sister relationship with Ippy virus in GPC gene tree, with the Lassa/Gbagroube/Mopeia/Mobala virus clade in NP gene tree, and with Mobala virus in L gene tree (Fig. 2). Taken together, though the precise phylogenetic position of Menekre virus could not be determined, it appears to belong to the group of presumably apathogenic African OWAs. A sequence feature supporting this conclusion is the presence of a second stem-loop structure in the intergenic region of S RNA of Menekre virus, which otherwise occurs only in Mopeia virus [20].

**Figure 3:**
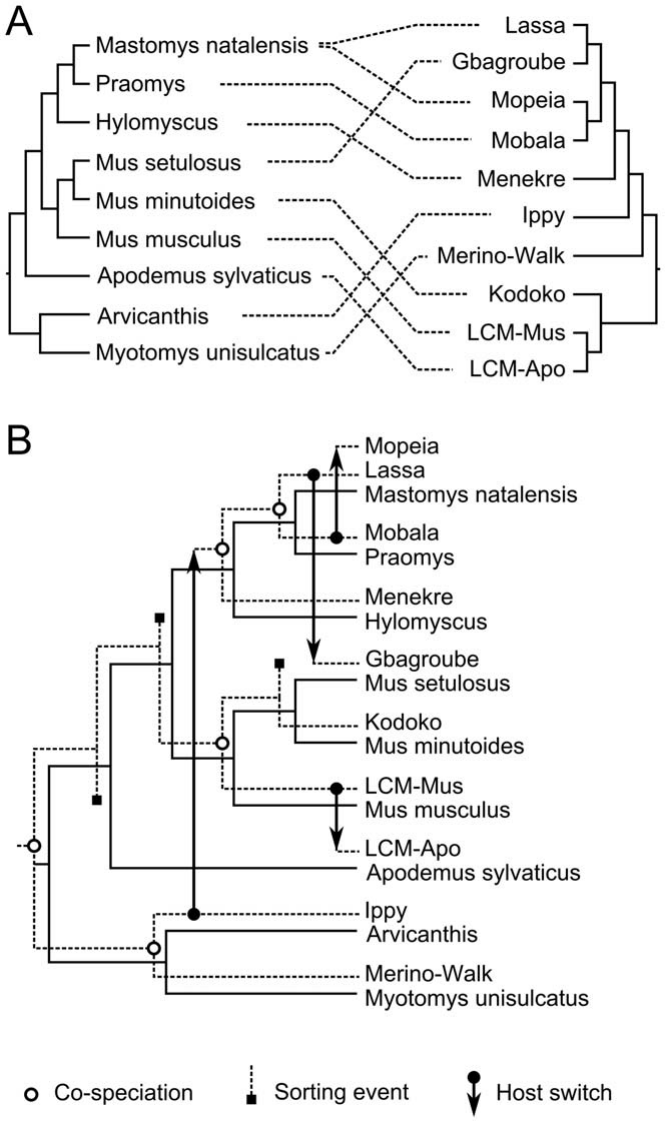
Historical associations between Old World arenaviruses and their rodent hosts. (A) Juxtaposition of host and virus tree. The rodent tree was compiled from published data [23], [24], [25], [26], [27]. The positions of Gbagroube and Menekre virus in the virus tree correspond to those in the NP gene tree shown in Fig. 2. Primary virus–host associations are indicated by dotted lines [2], [3], [4], [5], [6], [8], [28], [29]. (B) Reconciliation of virus tree (solid lines) with host tree (dotted lines) by CoRe-PA 0.3 [22]. One of several best-fit scenarios proposed by the program is shown, which includes 5 co-speciations, 4 host-switches, and 3 sorting events [30].

The identification of arenaviruses in rodents so far not implicated as OWA host species led us to investigate how the new data fit with current hypotheses on arenavirus–host co-evolution [1], [21]. The phylogenies of the OWAs were compared and reconciled with those of their hosts using the CoRe-PA 0.3 program [22]. As the virus phylogeny calculated with NP gene sequences was most stable and inferred by both BEAST and PhyML, it was used for the co-phylogeny analysis. The host tree was compiled from literature data [23], [24], [25], [26], [27]. All rodents known to be associated with an OWA were included [2], [3], [4], [5], [6], [8], [28], [29]. Juxtaposition of virus and host trees showed no obvious congruence between them (Fig. 3A). Reconciliation of the virus tree with the host tree revealed that a maximum of 5 co-speciation events (joint divergence of both host and virus) might have happened during evolution. The maximum of 5 co-speciations was found by CoRe-PA irrespective of whether a pre-defined cost model was used or optimal co-phylogenies were searched for with automatically calculated cost values. In addition to 5 co-speciations, best-fit scenarios included 4 host switches (transfer of virus from one host to another host that is not itself the immediate descendant of the source host) and 3 sorting events (apparent absence of virus in the descendants of a host that had previously been associated with virus) [30]. One of several possible co-phylogeny scenarios is shown in Fig. 3B. To test if the observed frequency of 5 co-speciations is biologically meaningful, randomization tests implemented in CoRe-PA were performed. Reconciliation of randomized virus trees with the host tree showed that 5 or more co-speciations occur just by chance in 63% of the random co-phylogenies; the average was 4.8 events. Thus, co-speciation between OWAs and their respective hosts — the hallmark of co-evolution — was not observed at a frequency above statistical noise. In conclusion, the currently available data do not lend support to the hypothesis that arenaviruses co-evolved with their hosts in Africa. Host-switching and adaptation might have been involved in evolution.

**Figure 4:**
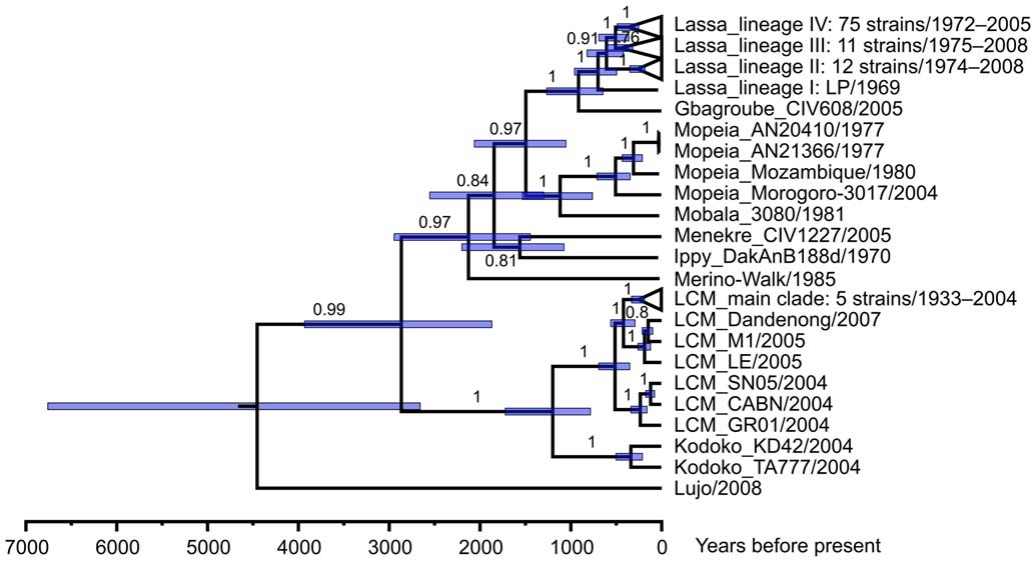
Timing of speciation events in the Old World arenavirus clade. Phylogenies were inferred by BEAST software based on 122 partial nucleotide sequences of NP gene of viruses collected over a period of 70 years. The substitution rate was estimated from the data set. Posterior values are indicated on the branches. Blue bars indicate the smallest interval that contains 95% of the posterior probability density for a node age, i.e. the time to the most common recent ancestor of the respective clade. The year of collection is shown with the strains. The complete list of taxa with GenBank accession numbers and year of collection is provided in Table S1.

Another piece of information on the evolutionary history of arenaviruses in Africa may be obtained by estimating the nucleotide substitution rate. The alignment of 122 partial NP sequences from OWAs, mainly collected between 1969 and 2008, contained sufficient temporal structure to estimate a global rate for the clade. The BEAST program calculated a rate of 5.1×10^−4^ substitutions×site^−1^×year^−1^ with good statistical precision: the smallest interval that contains 95% of the posterior probability density was 3.6–6.8×10^−4^ site^−1^×year^−1^. Nevertheless, it may be interpreted with caution, given that the difference in date between the samples is not large compared to the overall time scale of the reconstruction. The rate estimate for the OWA clade is in the same order of magnitude than estimates for other RNA viruses [31], [32]. It implies that the ancestors of contemporary OWAs are between 3000 and 7000 years old (Fig. 4). Speciation of Menekre and Gbagroube virus was calculated to have happened within the past 2000 years. All these times are orders of magnitudes too recent to match with the speciation times of the respective rodent hosts some millions of years ago [23], [25], [33].

## Discussion

Screening of about 1200 small mammals of various genera from Côte d'Ivoire provided genetic evidence for the existence two new arenavirus species, Menekre and Gbagroube virus, in *Hylomyscus* sp. and *Mus (Nannomys) setulosus*, respectively. The serological data are consistent with the circulation of Gbagroube virus in *Mus (Nannomys) setulosus* around the Gbagroube village. No evidence for the spread of this virus in a larger geographical area was found. In addition, the data from the other animal species do not suggest the presence of major hot-spots of arenavirus circulation in the study area. Only few animals have been found serologically positive. Noteworthy is a shrew (*Crocidura* sp.) that was captured on the same trapping line like the virus-positive *Mus (Nannomys) setulosus*, which raises the hypothesis of a cross-species transmission of Gbagroube virus from the rodent to the shrew. The arenavirus antibodies in *Mus (Nannomys) minutoides*, reaching a prevalence of nearly 10%, may point to the circulation of Kodoko virus, which has been found in this rodent species in the neighboring Guinea [5]. The lack of Lassa virus detection by PCR and the low seroprevalence of arenavirus antibodies (0.7%) in *Mastomys natalensis* suggest that Lassa virus is either absent (i.e. the antibodies result from infection with another arenavirus) or circulating at extremely low level in south, east, and middle west of Côte d'Ivoire. In 2002, the study sites were selected in areas with high rainfall in Côte d'Ivoire based on the assumption that rainfall is an important determinant for Lassa virus circulation (the Lassa virus endemic areas Liberia, Guinea, and Sierra Leone are characterized by high rainfall; see map at http://www.cartographie.ird.fr/images/pluvio_afrique/pluvio1.gif). However, recent risk mapping using a larger set of environmental conditions and sophisticated mathematical modeling predicts Lassa virus circulation rather in the west and north of Côte d'Ivoire [12], which is compatible with the detection of Lassa virus in the South of Mali [13], [14]. Further studies in Côte d'Ivoire are needed to clarify whether Lassa virus is endemic in the country or not.

Related studies in Africa found two new arenaviruses (F4-8/TZA and TZ22285/TZA) in Tanzania by genetic screening of 600 rodents [7] and one new arenavirus (Kodoko) in Guinea by genetic screening of 1500 animals [5]. Including our study, testing of about 3300 rodents from three localities in Africa resulted in the genetic identification of five new arenaviruses. Extrapolating this number in view of the great diversity and quantity of rodents on the whole African continent, it is tempting to speculate that still a considerable number of undescribed arenaviruses circulate in Africa. Some of them may have the potential to cause human disease as exemplified by Lujo virus which emerged so far one time causing several fatalities [10], [11]; its host is still obscure. Whether Menekre and Gbagroube virus are able to infect humans and have pathogenic potential, is not known. In contrast to Lassa virus hosted by the commensal rodent *Mastomys natalensis*, they are associated with less abundant rodent species, which do not live as close to humans. However, *Hylomyscus* sp. and *Mus (Nannomys) setulosus* may have contact to humans as they occur in cultivation areas. Thus, provided they can cause disease, cases might be observed rarely.

From a technical point of view, our previously described screening PCR assay targeting the L gene has proven a powerful molecular tool for discovery of new arenaviruses [17]. It led to the discovery of all the new arenaviruses in the rodent projects mentioned above. The technological base for molecular identification and characterization of unknown OWAs was further improved here by designing sets of OWA consensus primers targeting GPC and NP. These primers facilitated complete sequencing of the 3.5-kb S RNA segment of Menekre and Gbagroube virus from a few microliters of blood. The new sets of primers may prove useful in sequencing the S RNA of unknown arenaviruses in future, in particular if only limited amount of specimen is available. This also concerns F4-8/TZA, TZ22285/TZA, and Kodoko virus, which so far have neither been isolated nor sequenced on larger parts of the genome [5], [7]. We tried different cell cultures as well as newborn mice or transgenic mice with deficiencies in the cellular and innate immune response to isolate Menekre and Gbagroube virus. The amounts of available blood and tissue were extremely small, and thus, the failure to isolate them might have been due to the low quantity of inoculum. Technical reasons (e.g. degradation) or biological features of the viruses (e.g. receptor specificity) are not excluded as well. Nevertheless, the S RNA sequences obtained directly from the specimens contained sufficient information for taxonomic classification of the viruses and thorough phylogenetic studies.

While the phylogenetic position of Menekre virus is somewhat ambiguous — it may stabilize with the availability of sequences of new arenaviruses related to Menekre virus — the position of Gbagroube virus is quite clear and interesting. This virus is now the closest relative of Lassa virus. In the GPC amino acid sequence, Gbagroube virus shows nearly the same genetic distance to some Lassa virus strains (9.8% between Gbagroube virus and Lassa virus Nig08-04) than some Lassa virus strains among themselves (9.2% between Lassa virus strains CSF and Nig08-A47). However, Gbagroube virus probably is not a spillover infection of Lassa virus in Côte d'Ivoire or vice versa. In the phylogeny, it is basal to the Nigerian Lassa virus strains (lineages I to III) and phylogenetically far from the Lassa virus strain circulating in the area of Côte d'Ivoire (strain AV in lineage IV). Assuming that the dating of evolutionary events as shown in Figure 4 is roughly correct, the most recent common ancestor of the Gbagroube and Lassa virus clades has split into the ancestors of the Nigerian Lassa virus lineages and Gbagroube virus about 1000 years ago, while Lassa virus diversified into the lineage IV strains, including strain AV, within the past 500 years. The close relationship of Gbagroube virus to the basal Lassa virus strains raises the question of whether Gbagroube virus should be considered a basal strain of Lassa virus. According to the current species-differentiating cut-off value of 12% amino acid difference in NP [19], it is not Lassa virus. However, this limit may be extended in future along with the availability of new virus sequences.

Reconstructing the evolutionary history of viruses and timing of diversification events has become an intensively studied issue in recent years. Early hypotheses for evolution of rodent-borne viruses such as arena- and hantaviruses assumed that these viruses co-evolve with their hosts [1], [34], which implies that the viruses are as old as their hosts, more than 20 Mio years [25], [33]. These hypotheses were based on the association of New World and Old World viruses with New World and Old World rodents, respectively, and a certain level of congruence between virus and host phylogenetic trees. However, with the growing number of known virus species, the congruence between virus and host phylogenies decreased rather than increased. In a recently published co-phylogenetic reconciliation study, the authors could not provide evidence for co-divergence between hantaviruses and their hosts [35]. In our study, by including the novel sequences from Côte d'Ivoire, we show there is no congruence between OWA and host phylogeny that exceeds the level of statistical noise. While this is not direct evidence against the co-evolution hypothesis, it eliminates in part the factual basis for this hypothesis. Our analysis suggests that host-switching may have contributed to arenavirus evolution in Africa, independent of the timing of these events.

How old viruses are, is a controversial issue. Estimates of the molecular clock rate based on sequences of contemporary RNA viruses are typically in the range of 10^−4^ to 10^−3^ substitutions×site^−1^×year^−1^ [31], [32], as we found here for the OWA clade. These rates imply that the speciation of RNA viruses took place some 1,000 or 10,000 years ago — far too recent to be compatible with a co-evolution concept. These estimates are challenged by the discovery of negative strand RNA virus sequences (filo- and bornaviruses) that had been integrated into host genomes some millions of years ago [36], [37], [38], [39]. The long-term evolution of RNA viruses over millions of years apparently is much slower than the short-term evolution as observed with contemporary viruses, a paradox for which there is no good explanation [40], [41]. A long-term evolutionary history may explain the association of New World and Old World arenaviruses with New World and Old World mice, respectively, which remained stable due to the geographical constraints. However, within the African continent the signatures of co-evolution may have been obliterated by multiple host-switching and lineage extinction events.

## Materials and Methods

### Ethics statement

Permission to sample small mammals has been granted by the Ministère Délégué auprès du Ministre de la Solidarité Chargé de la Santé, République de Côte d'Ivoire (permit no. 1155 MDCS/CAB-1/kss to the Director of the Institut Pasteur, Côte d'Ivoire, signed by le directeur de cabinet, Abidjan, 19 December 2002).

### Wildlife trapping

Traps were placed in the fields and the houses of 9 villages in south, east, and middle west of Côte d'Ivoire (Table 1). Trapping sessions were performed in December 2003; March, November, and December 2004; March, August, October, and November 2005. Animals were taxonomically classified according to morphological criteria (weight, length of head and body, length of tail, length of hind food, length of the ear, color etc.) and dissected in the field. Blood and organ samples were stored frozen, and voucher specimens were deposited at the Museum National d'Histoire Naturelle, Paris.

### RT-PCR and sequencing

Blood of 3 animals was pooled (3×20 µl; for *Mus (Nannomys)* 3×5 µl) and RNA was prepared by using the E.Z.N.A. Blood RNA Kit (PEQLAB). Screening was performed by using two pan–OWA RT-PCRs. The first assay targeting the large (L) gene was performed as described [17]. The second assay targeting the glycoprotein precursor (GPC) gene was performed using primers OWS-1-fwd (GCGCACCGGGGATCCTAGGC) and OWS-1000A-rev (AGCATGTCACAAAAYTCYTCATCATG), and the QIAGEN OneStep RT-PCR Kit (Qiagen). All pools were also tested by a Lassa virus-specific RT-PCR as described [42]. RT-PCRs specific for Menekre and Gbagroube virus sequences, respectively, were based on a diagnostic Lassa virus RT-PCR assay [43]; just the reverse primer was modified to match to Menekre virus (MenS-339-rev GTTTTTGCTGCAAGATAAAGGCATGGTCAC) or Gbagroube virus (GbaS-339-rev GTTTTTCGTACAAGAGAGCGGCATGGTCAT). Samples were tested individually rather than pooled in the virus-specific RT-PCRs. For sequencing the complete S RNA segment, nucleoprotein (NP) gene fragments were amplified using the SuperScript One-Step RT-PCR Kit (Invitrogen) and the pan-OWA primer mixtures (i) OWS-2165A/B-fwd (TCTTCAGGTCTCCCTTCWATGTCNATCCANGT+TCTTCAGGTCTCCCTTCWATGTCNATCCA) and OWS-2840A/B-rev (AAYAAYCAGTTTGGGACNATGCCAAG+AAYAAYCAGTTTGGGACNATGCC) or (ii) OWS-2380A/B-fwd (GATGTYCTTGATGCWATGTATGGCCANCC+GATGTYCTTGATGCWATGTATGGCCA) and OWS-2840A/B-rev. PCR fragments were sequenced on both stands. Remaining gaps were closed after designing ≈50 virus-specific primers. S RNA sequences (excluding the conserved 19 nucleotides at the termini) were assembled using SeqMan software (DNASTAR).

### Phylogenetic analysis

Phylogenetic analysis included the novel sequences as well as OWA sequences available from GenBank by January 2011. Amino acid sequences of GPC, NP, and L gene were aligned by using MacVector software (MacVector). The alignment was refined manually and used for calculation of *p*-distance values. The corresponding nucleotide sequences were aligned manually guided by the amino acid alignment. Recombination events within the S RNA segment were not detected by RDP3 software [44]. FindModel (http://www.hiv.lanl.gov/content/sequence/findmodel/findmodel.html) identified the general time-reversible model of sequence evolution with a gamma distribution of among-site nucleotide substitution rate variation (GTR+gamma) as the substitution model that best describes the data in the alignments; it was used in all phylogenetic analyses. The fraction of invariant sites was not considered because estimates for this parameter are very sensitive to the number of taxa. Phylogenies were inferred by the Bayesian Markov Chain Monte Carlo method implemented in BEAST software [45] using nucleotide sequence alignments of complete GPC gene (42 taxa, 1518 sites), complete NP gene (41 taxa, 1728 sites), and partial L gene (78 taxa, 342 sites) (see Table S1 for the list of taxa and GenBank accession numbers) with the following parameters: GTR+gamma model with strict clock (mean substitution rate fixed at 1); constant population size; 10^7^ steps with sampling every 10^5^th step; and two independent runs combined (effective sampling size >200 for all parameters). For complete genes, the tree topology was reexamined by a maximum likelihood approach with bootstrap testing implemented in the PhyML program [46] with the parameters: GTR+gamma model and consensus of 500 bootstrap trees.

As partial NP gene sequences were available for a large number of OWA strains isolated over a period of more than 70 years, the respective alignment (122 taxa, 640 sites) contained sufficient temporal structure to estimate substitution rates and divergence times. The list of taxa with GenBank accession numbers and references for the collection dates is provided in Table S1. The NP gene alignment was analyzed with BEAST under the assumption of a GTR+gamma model with strict molecular clock, constant population size, and a substitution rate estimated from the data set. Four independent runs with 10^7^ steps and sampling every 10^5^th step were combined (effective sampling size >100 for all parameters).

Co-phylogeny scenarios were reconstructed by using CoRe-PA 0.3 [22] software with pre-defined cost values (co-speciation, −2; sorting event, 1; duplication, 2; host switch, 2) or automatically calculated cost values. Randomization testing was performed with CoRe-PA using standard parameters and the above pre-defined cost values. The virus tree was randomized under the beta = −1 model.

### Rodent genotyping

Rodents were genotyped by amplification and sequencing the mitochondrial genes for 16S rRNA (∼530 nucleotides) using primers16Sar L (CGCCTGTTTAACAAAAACAT) and 16S-Hm (AGATCACGTAGGACTTTAAT) and cytochrome B (∼1190 nucleotides) using primers L7 (ACCAATGACATGAAAAATCATCGTT) and H15915 (TCTCCATTTCTGGTTTACAAGAC) [47], [48]. The genes were subjected to BLAST search (http://blast.ncbi.nlm.nih.gov/) or aligned with rodent sequences from GenBank of the superfamily Muroidea. Phylogenetic reconstruction was performed by using the PhyML program [46].

### Rodent serology

Lassa virus-infected cells were spread onto immunofluorescence slides, air-dried, and acetone-fixed. Rodent serum was diluted 1∶20 in phosphate-buffered saline (PBS). If serum was not available in sufficient quantities, rodent organs (heart and spleen) were vigorously washed in 100 µl PBS and the wash fluid was used for the assay. Organ wash fluid had previously been validated for antibody detection and shown to be equivalent to serum (E. Fichet-Calvet, unpublished). Diluted serum or organ wash fluid was incubated with the cells and bound rodent IgG was detected with anti-mouse IgG-fluorescein isothiocyanate (Dianova). Signals were visualized with a fluorescence microscope.

### Virus isolation attempts

Virus isolation was attempted with blood and spleen specimens of rodents CIV839, CIV843, CIV1227, CIV608, CIV674, and CIV1290. About 8 mm^3^ of spleen was homogenized in 1 ml cell culture medium using a bead mill. The homogenate was cleared by centrifugation. African green monkey kidney (Vero) cells and mouse fibroblast cells (L cells) in culture vials were inoculated with blood and cleared homogenate, respectively. Cells and supernatant were passaged 3 times. Potential virus growth was monitored in supernatant by pan-OWA RT-PCR (see above), and in cells by immunofluorescence using a mixture of OWA-specific monoclonal antibodies [49]. About 700 µl spleen homogenate was also inoculated intraperitoneally into mice lacking a functional interferon type I system as well as mature B and T cell (AR129 mice). Blood samples were taken on days 4, 7, 10, 13, and 20 post inoculation. In addition, newborn mice were inoculated intracerebrally with about 20 µl of spleen homogenate. Sera from AR129 mice and brain homogenate of newborn mice were tested by pan-OWA RT-PCR and used to inoculate Vero and BHK-21 cells. Potential virus growth was monitored as described above.

## Supporting Information

Table S1 Old World arenavirus strains included in the study. The table provides GenBank accession numbers for all sequences included in the phylogenetic analyses as well as collection dates with references for the NP gene sequences.
(PDF)

Abstract S1 Translation of the abstract into French by Elisabeth Fichet-Calvet.
(PDF)
