# Supplementary material for: Novel Arenavirus Sequences in Hylomyscus sp. and Mus (Nannomys) setulosus from Côte d'Ivoire: Implications for Evolution of Arenaviruses in Africa
Source: PLoS One. 2011 Jun 9;6(6):e20893. doi: 10.1371/journal.pone.0020893 (PMC3111462; doi:10.1371/journal.pone.0020893)
Supplement: Table S1 — Old World arenavirus strains included in the study. The table provides GenBank accession numbers for all sequences included in the phylogenetic analyses as well as collection dates with references for the NP gene sequences. (PDF) [file pone.0020893.s001.pdf]

Supplementary Table S1. Old World arenavirus strains included in the study.

Virus strain	GPC GenBank accession no.	L GenBank accession no.	NP GenBank accession no.	NP Year of collection	NP Reference for collection date
SL_Josiah	J04324	U63094	J04324*	1976	[1]
SL_NL	AY179173	AY179172	AY179173*	2000	[2]
SL_06-2057		GU979507			
SL_15		AY363906			
SL_20		AY363907			
SL_21		AY363908			
SL_25		AY363909			
SL_26		AY363910			
SL_620		AY363905			
SL_801100			AF182253	1978	[3]
SL_801101			AF182248	1978	[3]
SL_801102			AF182251	1978	[3]
SL_801103			AF182249	1978	[3]
SL_801104			AF182241	1978	[3]
SL_801105			AF182239	1978	[3]
SL_801106			AF182242	1978	[3]
SL_801107			AF182240	1978	[3]
SL_801108			AF182244	1978	[3]
SL_801109			AF182243	1978	[3]
SL_801618			AF182234	1979	[3]
SL_802662			AF182233	1980	[3]
SL_803205			AF182235	1976	[3]
SL_803206			AF182250	1976	[3]
SL_803209			AF182236	1975	[3]
SL_803211			AF182247	1972	[3]
SL_803972			AF182256	1982	[3]
SL_806827			AF182238	1977	[3]
SL_806843			AF182254	1993	[3]
SL_807875			AF182245	1982	[3]
SL_807974			AF182257	1976	[3]
SL_807976			AF182246	1977	[3]
SL_9607290			AF182237	1996	[3]
SL_9607300			AF182252	1996	[3]
SL_9607302			AF182255	1996	[3]
LIB_04-2739		AY870334			
LIB_05-236/88		GU979512			
LIB_05-1580/121		GU979511			
LIB_05-2096/127		GU979510			
LIB_05-2406/129		GU979509			
LIB/GUI_Macenta = 806828	AY628201	AY628200	AY628201*	1981	[3,4]
LIB_Z148 = 803793	AY628205	AY628204	AY628205*	1980	[3,4]
LIB_803201			AF182259	1972	[3]
LIB_803203			AF182266	1972	[3]
LIB_803204			AF182258	1972	[3]
LIB_803791			AF182262	1980	[3]
LIB_803792		AY693638	AF182260	1980	[3]
LIB_803796			AF182271	1981	[3]
LIB_806829			AF182264	1981	[3]
LIB_807977			AF182268	1981	[3]
GUI_807868			AF182269	1996	[3]
GUI_807992			AF182267	1997	[3]
GUI_807998			AF182265	1997	[3]
GUI_808255			AF182270	1996	[3]
GUI_BA263			DQ832669	2003	[5,6]
GUI_BA289			DQ832670	2003	[5,6]
GUI_BA302			DQ832671	2003	[5,6]
GUI_BA350			DQ832672	2003	[5,6]
GUI_BA354			DQ832673	2003	[5,6]
GUI_BA356			DQ832674	2003	[5,6]

Supplementary Table S1 (cont.)

Virus strain	GPC GenBank accession no.	L GenBank accession no.	NP GenBank accession no.	NP Year of collection	NP Reference for collection date
GUI_BA366	GU830839	GU979513	GU830839*	2003	[5,6]
GUI_BA375			DQ832676	2003	[5,6]
GUI_BA377			DQ832677	2003	[5,6]
GUI_BA378			DQ832678	2003	[5,6]
GUI_BA382			DQ832679	2003	[5,6]
GUI_BA384			DQ832680	2003	[5,6]
GUI_BA686			DQ832681	2004	[5,6]
GUI_BG129			DQ832667	2003	[5,6]
GUI_GB132			DQ832668	2003	[5,6]
GUI_DGD104			DQ832698	2005	[5,6]
GUI_DGD112			DQ832699	2005	[5,6]
GUI_DGD13			DQ832693	2005	[5,6]
GUI_DGD28			DQ832694	2005	[5,6]
GUI_DGD35			DQ832695	2005	[5,6]
GUI_DGD4			DQ832692	2005	[5,6]
GUI_DGD43			DQ832696	2005	[5,6]
GUI_DGD87			DQ832697	2005	[5,6]
GUI_TA341			DQ832683	2003	[5,6]
GUI_TA416			DQ832682	2003	[5,6]
GUI_TA444			DQ832684	2003	[5,6]
GUI_TA462			DQ832685	2003	[5,6]
GUI_TA464			DQ832686	2003	[5,6]
GUI_TA471			DQ832687	2003	[5,6]
GUI_TA491			DQ832688	2003	[5,6]
GUI_TA817			DQ832689	2004	[5,6]
GUI_TA820			DQ832690	2004	[5,6]
GUI_TA846			DQ832691	2004	[5,6]
IC_AV	AF246121	AY179171	AF246121*	2000	[2]
NIG_04-10		AY693637			
NIG_05-043		GU481057			
NIG_05-SE40		GU481059			
NIG_07-05		GU481062			
NIG_08-02		GU481065			
NIG_08-03		GU481067			
NIG_08-04	GU481068	GU481069	GU481068*	2008	[7]
NIG_08-A18	GU481070	GU481071	GU481070*	2008	[7]
NIG_08-A19	GU481072	GU481073	GU481072*	2008	[7]
NIG_08-A37	GU481074	GU481075	GU481074*	2008	[8]
NIG_08-A41	GU481076	GU481077	GU481076*	2008	[8]
NIG_08-A47	GU481078	GU481079	GU481078*	2008	[8]
NIG_803208			AF182232	1975	[3]
NIG_803210			AF182229	1975	[3]
NIG_803212			AF182230	1976	[3]
NIG_803213	AF181854	AY693640	AF181854*	1974	[3]
NIG_803214			AF182220	1974	[3]
NIG_806316			AF182221	1989	[3]
NIG_806319			AF182224	1989	[3]
NIG_806320			AF182223	1989	[3]
NIG_806321			AF182225	1989	[3]
NIG_806322			AF182226	1989	[3]
NIG_806791		AY693639	AF182228	1993	[3]
NIG_807975			AF182231	1976	[3]
NIG_808031			AF182222	1994	[3]
NIG_9608911			AF182272	1996	[3]
NIG_CSF	AF333969	AY179174	AF333969*	2000	[3]
NIG_GA391	X52400		X52400*	1977	[3]
NIG_LP	AF181853		AF181853*	1969	[3]
NIG_Weller = 803787	AY628206		AY628206*	1981	[3,4]
Gbagroube_CIV608	GU830848	GU830849	GU830848*	2005	This study

Supplementary Table S1 (cont.)

Virus strain	GPC GenBank accession no.	L GenBank accession no.	NP GenBank accession no.	NP Year of collection	NP Reference for collection date
Gbagroube_CIV674		GU830852			
Gbagroube_CIV1290		GU830855			
Menekre_CIV1227	GU830862	GU830863	GU830862*	2005	This study
Menekre_CIV839		GU830858			
Menekre_CIV843		GU830861			
Mopeia_AN20410	AY772170	AY772169	AY772170*	1977	[9]
Mopeia_AN21366	M33879	AY363904	M33879*	1977	[9]
Mopeia_Mozambique	DQ328874	DQ328875	DQ328874*	1980	[10]
Mopeia_Morogoro-18		EU914110			
Mopeia_Morogoro-30		EU914107			
Mopeia_Morogoro-50		EU914109			
Mopeia_Morogoro-51		EU914108			
Mopeia_Morogoro-3016		EU914122			
Mopeia_Morogoro-3017	EU914103	EU914104	EU914103*	2004	[11]
Mopeia_Morogoro-3101		EU914121			
Mopeia_Morogoro-3115		EU914120			
Mopeia_Morogoro-3124		EU914119			
Mopeia_Morogoro-3160		EU914118			
Mopeia_Morogoro-3164		EU914117			
Mopeia_Morogoro-3177		EU914116			
Mopeia_Morogoro-3220		EU914115			
Mopeia_Morogoro-3227		EU914114			
Mopeia_Morogoro-3230		EU914113			
Mopeia_Morogoro-3235		EU914112			
Mopeia_Morogoro-3237		EU914111			
Mobala_3080	AY342390	DQ328876	AY342390*	1981	[12]
Mobala_3099		AY693641			
Ippy_DakAnB188d	DQ328877	AY363902	DQ328877*	1970	[13]
Merino Walk	GU078660	GU078661	GU078660*	1985	[14]
LCM_Armstrong	M20869	J04331	M20869*	1933	[15]
LCM_WE	M22138	AF004519	M22138*	1934	[16,17]
LCM_CH-5692	AF325214	AY363903	AF325214*	1999	[18]
LCM_CH-5871	AF325215		AF325215*	2000	[18]
LCM_LE			EF164923	2005	[19]
LCM_M1	AB261991		AB261991*	2005	[20]
LCM_Marseille#12	DQ286931	DQ286932	DQ286931*	2004	[21]
LCM_Dandenong	EU136038	EU136039	EU136038*	2007	[22,23]
LCM_MX	EU195888	EU195889	Y16308*		
LCM_Pasteur	DQ868485	DQ868486	DQ868485*		
LCM_Traub	DQ868487	DQ868488	DQ868487*		
LCM_UBC-aggressive/docile	EU480450	EU480453	EU480450*		
LCM_Y	DQ118959				
LCM_GR01	FJ895883		FJ895883*	2004	[24]
LCM_CABN	FJ895882		FJ895882*	2004	[24]
LCM_SN05	FJ895884		FJ895884*	2004	[24]
Kodoko_KD42		EF179865	EF189587	2004	[25]
Kodoko_TA777		EF179864	EF189586	2004	[25]
F4-8/TZA/2008		GU182412			
TZ22285/TZA/2008		GU182413			
Lujo	FJ952384	FJ952385	FJ952384*	2008	[26]

The origin of Lassa virus strains is indicated by a prefix: SL, Sierra Leone; LIB, Liberia; GUI, Guinea; IC, Ivory Coast; NIG, Nigeria.
* Complete NP sequences.

References:

1. Auperin DD, Sasso DR, McCormick JB (1986) Nucleotide sequence of the glycoprotein gene and intergenic region of the Lassa virus S genome RNA. Virology 154: 155-167.
2. Schmitz H, Kohler B, Laue T, Drosten C, Veldkamp PJ, et al. (2002) Monitoring of clinical and laboratory data in two cases of imported Lassa fever. Microbes Infect 4: 43-50.
3. Bowen MD, Rollin PE, Ksiazek TG, Hustad HL, Bausch DG, et al. (2000) Genetic diversity among Lassa virus strains. J Virol 74: 6992-7004, and references therein.
4. Jahrling PB, Frame JD, Smith SB, Monson MH (1985) Endemic Lassa fever in Liberia. III. Characterization of Lassa virus isolates. Trans R Soc Trop Med Hyg 79: 374-379.
5. Lecompte E, Fichet-Calvet E, Daffis S, Koulemou K, Sylla O, et al. (2006) Mastomys natalensis and Lassa fever, West Africa. Emerg Infect Dis 12: 1971-1974.
6. Fichet-Calvet E (2010) personal communication.
7. Ehichioya DU, Hass M, Olschlager S, Becker-Ziaja B, Onyebuchi Chukwu CO, et al. (2010) Lassa fever, Nigeria, 2005-2008. Emerg Infect Dis 16: 1040-1041.
8. Ehichioya DU, Hass M, Becker-Ziaja B, Ehimuan J, Asogun DA, et al. (2011) Current molecular epidemiology of Lassa virus in Nigeria. J Clin Microbiol: in press.
9. Wulff H, McIntosh BM, Hamner DB, Johnson KM (1977) Isolation of an arenavirus closely related to Lassa virus from Mastomys natalensis in south-east Africa. Bull World Health Organ 55: 441-444.
10. Johnson KM, Taylor P, Elliott LH, Tomori O (1981) Recovery of a Lassa-related arenavirus in Zimbabwe. Am J Trop Med Hyg 30: 1291-1293.
11. Günther S, Hoofd G, Charrel R, Roser C, Becker-Ziaja B, et al. (2009) Mopeia virus-related arenavirus in natal multimammate mice, Morogoro, Tanzania. Emerg Infect Dis 15: 2008-2012.
12. Gonzalez JP, McCormick JB, Saluzzo JF, Herve JP, Georges AJ, et al. (1983) An arenavirus isolated from wild-caught rodents (Praomys species) in the Central African Republic. Intervirology 19: 105-112.
13. Digoutte JP (1978) Ippy (IPPY). Am J Trop Med Hyg 27: 389-390.
14. Palacios G, Savji N, Hui J, Travassos da Rosa A, Popov V, et al. (2010) Genomic and phylogenetic characterization of Merino Walk virus, a novel arenavirus isolated in South Africa. J Gen Virol 91: 1315-1324.
15. Armstrong C, Lillie RD (1934) Experimental lymphocytic choriomeningitis of monkeys and mice produced by a virus encountered in studies of the 1933 St. Louis encephalitis epidemic. Pub Health Rep 49: 1019-1027.
16. Scott TF, Rivers TM (1936) Meningitis in Man Caused by a Filterable Virus : I. Two Cases and the Method of Obtaining a Virus from Their Spinal Fluids. J Exp Med 63: 397-414.
17. Rivers TM, Scott TF (1936) Meningitis in Man Caused by a Filterable Virus : II. Identification of the Etiological Agent. J Exp Med 63: 415-432.
18. Asper M, Hofmann P, Osmann C, Funk J, Metzger C, et al. (2001) First outbreak of callitrichid hepatitis in Germany: genetic characterization of the causative lymphocytic choriomeningitis virus strains. Virology 284: 203-213.
19. Meritet JF, Krivine A, Lewin F, Poissonnier MH, Poizat R, et al. (2009) A case of congenital lymphocytic choriomeningitis virus (LCMV) infection revealed by hydrops fetalis. Prenat Diagn 29: 626-627.
20. Ike F, Bourgade F, Ohsawa K, Sato H, Morikawa S, et al. (2007) Lymphocytic choriomeningitis infection undetected by dirty-bedding sentinel monitoring and revealed after embryo transfer of an inbred strain derived from wild mice. Comp Med 57: 272-281.
21. Charrel RN, Retornaz K, Emonet S, Noel G, Chaumoitre K, et al. (2006) Acquired hydrocephalus caused by a variant lymphocytic choriomeningitis virus. Arch Intern Med 166: 2044-2046.
22. Anonymous (2007) Arenavirus, organ transplants - Australia (VIC). ProMED-mail Archive Number 20070423.1325.
23. Palacios G, Druce J, Du L, Tran T, Birch C, et al. (2008) A new arenavirus in a cluster of fatal transplant-associated diseases. N Engl J Med 358: 991-998.
24. Ledesma J, Fedele CG, Carro F, Lledo L, Sanchez-Seco MP, et al. (2009) Independent lineage of lymphocytic choriomeningitis virus in wood mice (Apodemus sylvaticus), Spain. Emerg Infect Dis 15: 1677-1680.
25. Lecompte E, ter Meulen J, Emonet S, Daffis S, Charrel RN (2007) Genetic identification of Kodoko virus, a novel arenavirus of the African pigmy mouse (Mus Nannomys minutoides) in West Africa. Virology 364: 178-183.
26. Briese T, Paweska JT, McMullan LK, Hutchison SK, Street C, et al. (2009) Genetic detection and characterization of Lujo virus, a new hemorrhagic fever-associated arenavirus from southern Africa. PLoS Pathog 5: e1000455.
