# Supplementary material for: Novel Arenavirus Sequences in Hylomyscus sp. and Mus (Nannomys) setulosus from Côte d'Ivoire: Implications for Evolution of Arenaviruses in Africa
Source: PLoS One. 2011 Jun 9;6(6):e20893. doi: 10.1371/journal.pone.0020893 (PMC3111462; doi:10.1371/journal.pone.0020893)
Supplement: Abstract S1 — Translation of the abstract into French by Elisabeth Fichet-Calvet. (PDF) [file pone.0020893.s002.pdf]

Novel arenavirus sequences in *Hylomyscus* sp. and *Mus (Nannomys) setulosus* from Côte d'Ivoire: Implications for evolution of arenaviruses in Africa

David Coulibaly-N'Golo et al.

Résumé

Cette étude présente la découverte de nouveaux arenavirus en Afrique, et son implication en terme évolutif. Entre 2003 et 2005, 1228 rongeurs et musaraignes, représentant 14 genres, ont été capturés dans 9 villages situés dans le sud-ouest, le sud et l'est de la Côte d'Ivoire. Les specimens ont été testés par une double RT-PCR ciblée sur le segment L d'une part et sur le segment S d'autre part. Ils ont aussi été testés par immunofluorescence. Les séquences de deux nouvelles espèces d'Arenavidae, Menekre et Gbagroube, ont été détectées chez *Hylomyscus* sp et chez *Mus* (*Nannomys*) *setulosus* respectivement. Grâce au test sérologique, une infection par les arenavirus a été détectée chez 32% (7/22) des *M.* (*Nannomys*) *setulosus* vivant à Gbagroubre. Le virus de Lassa n' a pas été trouvé, bien que 60% des captures étaient des *Mastomys natalensis*. L'ARN complet du segment S et partial du segment L des ces nouvelles espèces virales a été séquencé, puis intégré dans une analyse phylogénétique. Le virus Gbagroube est un taxon très proche de Lassa, tandis que le virus Menekre est proche du complexe Ippy/Mobala/Mopeia. Des scenarios de co-phylogénie entre les différents arenavirus et leurs hôtes sont discutés, suggérant de multiples transferts d'hôtes au cours de l'évolution sur le continent africain.
